# Differential Regulation of Human Treg and Th17 Cells by Fatty Acid Synthesis and Glycolysis

**DOI:** 10.3389/fimmu.2019.00115

**Published:** 2019-02-04

**Authors:** Deborah Cluxton, Andreea Petrasca, Barry Moran, Jean M. Fletcher

**Affiliations:** ^1^School of Biochemistry and Immunology, Trinity Biomedical Sciences Institute, Trinity College Dublin, Dublin, Ireland; ^2^School of Medicine, Trinity Biomedical Sciences Institute, Trinity College Dublin, Dublin, Ireland

**Keywords:** T cells, Th17 cells, Treg cells, immunometabolism, fatty acid synthesis, glycolysis (glycolytic pathway), immune modulation

## Abstract

In this study we examined the metabolic requirements of human T helper cells and the effect of manipulating metabolic pathways in Th17 and Treg cells. The Th17:Treg cell axis is dysregulated in a number of autoimmune or inflammatory diseases and therefore it is of key importance to identify novel strategies to modulate this axis in favor of Treg cells. We investigated the role of carbohydrate and fatty acid metabolism in the regulation of human memory T helper cell subsets, in order to understand how T cells are regulated at the site of inflammation where essential nutrients including oxygen may be limiting. We found that Th17 lineage cells primarily utilize glycolysis, as glucose-deprivation and treatment with rapamycin resulted in a reduction in these cells. On the other hand, Treg cells exhibited increased glycolysis, mitochondrial respiration, and fatty acid oxidation, whereas Th17 cells demonstrated a reliance upon fatty acid synthesis. Treg cells were somewhat reliant on glycolysis, but to a lesser extent than Th17 cells. Here we expose a fundamental difference in the metabolic requirements of human Treg and Th17 cells and a possible mechanism for manipulating the Th17:Treg cell axis.

## Introduction

Th17 cells are highly proinflammatory cells which have been implicated in the development and perpetuation of many autoimmune and inflammatory diseases, including rheumatoid arthritis, inflammatory bowel disease, psoriasis, and multiple sclerosis (1–3). In contrast, regulatory T (Treg) cells play a crucial role in maintaining immune tolerance to self-antigen as well as dampening the inflammatory immune response (4). A balance between immune regulation and inflammation is required to maintain optimal immunity and disruption of the Th17:Treg cell axis may contribute to disease. Therefore, skewing the Th17:Treg cell axis in favor of immune regulation by exploiting mechanisms of Th17 cell inhibition may combat inflammation and autoimmunity, and targeting cellular metabolism may be one way to modulate this axis (5, 6).

The carbohydrate metabolism of immune cells shifts between glycolysis and oxidative phosphorylation, depending on their activation state and the availability of surrounding nutrients. The transition from quiescence to activation results in greater metabolic demands. Aerobic glycolysis is induced within minutes after TCR activation via pyruvate dehydrogenase kinase 1 (PDHK1), which diverts pyruvate away from oxidative phosphorylation to lactate production independent of transcription, translation, or increased glucose uptake (7). Following activation, glycolysis involves increased glucose uptake, mammalian target of rapamycin (mTOR)-dependent pathways and a subsequent increase in the synthesis of essential biomolecules for cell growth (7, 8). Activated T cells can still utilize oxidative phosphorylation (9); however, glycolysis is required for the differentiation of specific effector T cell subsets. In mice, mTOR inhibition with rapamycin results in the decreased differentiation of Th17 cells and a subsequent increase in Treg cell generation (10, 11). Additionally, newly differentiated murine Th17 cells demonstrate increased glucose transporter 1 (Glut1) expression and glucose uptake, whilst Treg cells show a decrease in both, compared with Th1 cells (12). Murine Treg cells have been shown to express less mTOR complex 1 (mTORC1), which is known to promote glycolysis, and subsequently express high levels of AMP-activated protein kinase (AMPK), a protein which promotes oxidative phosphorylation as AMP levels increase (12). Additionally, the mTOR complexes have been shown to be differentially expressed in murine effector T cells, with Th17 and Th1 cells expressing mostly mTORC1, while Th2 cells predominantly express mTORC2 (13). In agreement with these findings, the deletion of the hypoxia-inducible factor 1α (HIF-1α), which is known to increase the expression of glycolytic enzymes (10) and promote glycolysis during low oxygen availability (14), was shown to promote FoxP3 expression and Treg cell differentiation, and conversely decrease Th17 cell differentiation (10, 15).

In addition to carbohydrate metabolism, fatty acid (FA) metabolism has key roles in regulating innate and adaptive immune responses. The enforced expression of carnitine palmitoyltransferase 1 (CPT1; transporter of FAs into the mitochondria in order to promote their oxidation) in macrophage cell lines results in decreased production of proinflammatory cytokines (16). In murine studies, newly differentiated Treg cells demonstrated the highest oxidation of the FA palmitate compared with other effector T cell subsets (12). Additionally, Treg cell differentiation was inhibited following treatment with etomoxir (inhibitor of CPT1), while Th17 cell differentiation was unaffected (12). As previously described, Treg cells have enhanced expression of AMPK, which is known to promote mitochondrial lipid oxidation and therefore could give rise to increased FA oxidation (FAO). Interestingly, the upregulation of fatty acid synthesis (FAS) correlates with a downregulation of FAO, demonstrating that these pathways are reciprocally linked (17). The inhibition of acetyl-CoA carboxylase (ACC) enzymes (involved in the FAS pathway) with soraphen A can decrease IL-17-expressing T cell differentiation, the expression of Th17 cell-specific genes, and IL-17 production by murine T cells under Th17-skewing conditions (18). Following induction of experimental autoimmune encephalomyelitis (EAE), mice with a T cell-specific knockdown of ACC1 showed no signs of disease. Moreover, these mice exhibited a marked reduction and promotion in the percentage of IL-17^+^ and FoxP3^+^ T cells respectively in the central nervous system compared with wild-type controls (18). Additionally, ACC1 is required in obesity for the DNA-binding activity of RORγt in differentiating Th17 cells, and therefore can regulate Th17 cell-mediated pathogenesis in disease (19). These studies highlight a requirement by murine Th17 cells for FAS, contrasted with the need for FAO by Treg cells during T cell differentiation. However, the metabolic pathways utilized by differentiated human Th cells at sites of inflammation is as of yet unknown. In this study, we investigated the role of carbohydrate and FA metabolism in the regulation of human memory Th cell subsets, in order to better understand how T cells are regulated.

## Materials and Methods

### Isolation of Human Cells

PBMC were isolated by Ficoll gradient centrifugation from leukocyte-enriched buffy coats from anonymous healthy donors (HC) (obtained with permission from the Irish Blood Transfusion Board, St. James's Hospital, Dublin and ethical approval from the School of Biochemistry and Immunology Research Ethics Committee, Trinity College Dublin). Total or memory (CD45RO^+^) CD4^+^ T cells were enriched using magnetic microbeads (Miltenyi Biotec). CD161^+^ (CD4^+^ CD45RO^+^ CD161^+^), CD161^−^ (CD4^+^ CD45RO^+^ CD161^−^), conventional T (Tconv; CD4^+^CD45RO^+^notCD25^+^CD127^Lo^), and Treg (CD4^+^CD25^+^CD127^Lo^) cells were sorted from HC PBMC on a MoFlo Legacy (Dako Cytomation/ Beckman Coulter) or FACSAria Fusion (BD Biosciences) cell sorter, with purities routinely >98%.

### T Cell Stimulation and Culture

Memory CD4^+^ T cells were cultured in complete RPMI (RPMI (Labtech) supplemented with 10% FCS (Sigma Aldrich), 2 mM L-glutamine with 1% penicillin/streptomycin (Sigma Aldrich) with irradiated antigen-presenting cells (irrAPC), anti-CD3 (eBioscience) and, where indicated, in the presence of dichloroacetate (DCA) (10 mM, Sigma Aldrich), rapamycin (20 nM, Sigma Aldrich), 5-(Tetradecyloxy)-2-furoic acid (TOFA) (1.2 μg/ml, Sigma Aldrich), C-75 (0.6 μg/ml, Sigma Aldrich), cerulenin (3.1 μM, Sigma Aldrich), or palmitate (25 μM, Seahorse Biosciences) for 5 days. Isolated CD161^+^, CD161^−^, and Tconv cells were cultured with irradiated antigen presenting cells (irrAPC), anti-CD3 and IL-2 (20 u/ml, eBioscience) for 6 days or in some cases with PMA/ionomycin for 18 h prior to Seahorse analysis. Isolated Treg cells were cultured using the Treg cell expansion kit (Miltenyi Biotec) in the presence of IL-2 (500 u/ml) for 6 days prior to Seahorse analysis. During glucose deprivation, memory CD4^+^ T cells were cultured in glucose-free complete RPMI (RPMI (Biosciences) supplemented with 10% dialysed FCS (Sigma Aldrich), 1% vitamin cocktail (InvivoGen), and 1% selenium/insulin cocktail (InvivoGen) supplemented with either 10 mM glucose (Sigma Aldrich) or 10 mM galactose (Sigma Aldrich) for 5 days.

### Flow Cytometry

Cells were labeled with the Fixable Viability Dye eFluor506 (eBioscience), then stained extracellularly with fluorochrome-conjugated antibodies specific for CD3, CD4, CD69, CD71, CD98, CD161, (BD Biosciences), CD8, CD25, CD127 (eBioscience), CD45RO (BioLegend), Glut1-GFP (5 μg/ml, Metafora Biosystems), MitoTracker Green (4 nM, InvivoGen), pS6 (Cell Signaling), and 2-NBDG (100 μM, InvivoGen). For FoxP3 (eBioscience) and/or pS6 analysis, cells were fixed and permeabilized for intranuclear staining (FoxP3 staining buffer kit, eBioscience). For intracellular cytokine analysis, cells were stimulated with PMA (50 ng/ml) and ionomycin (500 ng/ml) in the presence of brefeldin A (5 μg/ml, Sigma) for 5 h; then surface stained, fixed and permeabilized (Caltag fix and perm kit, Biosciences) and stained intracellularly for IL-17 (eBioscience) and IFN-γ (BD Biosciences). PMA and ionomycin stimulation of human CD4^+^ T cells down-regulates the expression of CD4, therefore CD4^+^ T cells were alternatively identified as CD3^+^CD8^−^. For proliferation of cells, the cells were stained intracellularly with Ki67 (BD Biosciences). Cells were acquired on a FACSCanto II or LSRFortessa cytometer (BD Biosciences) and analyzed using FlowJo software (FlowJo LLC). Viable lymphocytes were identified by forward and side scatter, with dead cells (Fixable Viability Dye eFluor506 positive) and doublets subsequently excluded. IrrAPC were stained with CellTrace CSFE and excluded from the analysis. Gates were set using fluorescence minus one (FMO) controls, isotype controls or unstimulated cells as appropriate.

### Metabolic Marker Expression and Glucose Uptake

For the examination of Glut1 expression, cells were washed with warmed Glut1 staining buffer A (complete RPMI, 0.2% EDTA, 2% sodium azide) and stained with Glut1-GFP (5 μg/ml, Metafora Biosystems) and surface markers in staining buffer A and incubated for 20 min at 37 °C. The cells were then washed with the Glut1 staining buffer B (PBS, 0.2% EDTA, 2% sodium azide, 2% FCS) and analyzed by flow cytometry.

For investigating glucose uptake, cells were washed twice in glucose-free RPMI and incubated at 37 °C for 15 min. Cells were incubated with 2-NBDG (100 μM) in glucose-free RPMI for 1 h at 37 °C, washed in pre-cooled PBS and stained for surface markers as previously described. Unfixed cells were then analyzed immediately by flow cytometry.

### Metabolic Flux Analysis

Extracellular acidification rate (ECAR) and oxygen consumption rate (OCR) were measured with an XF24 or XFe96 extracellular flux analyzer (Seahorse Bioscience). T cells were stimulated with PMA and ionomycin for 24 h prior to analysis by the Seahorse analyzer (6 x 10^5^ cells/well) and adhered to the plate with Cell-Tak (1 μg/well, Corning). ECAR and OCR were measured in real time following injections of oligomycin A (1 μg/ml), FCCP (450 nM), antimycin A (2.5 μM) and rotenone (500 nM), and 2-deoxyglucose (25 mM) (Sigma Aldrich).

FAO was measured using the XF Palmitate-BSA FAO Substrate (Seahorse Bioscience) using the manufacturer-recommended protocols. T cells were stimulated with PMA and ionomycin for 24 h prior to analysis by the Seahorse analyzer (6 × 10^5^ cells/well) and adhered to the plate with Cell-Tak. Cells were washed in substrate-limited medium (DMEM supplemented with 0.5 mM glucose, 1 mM L-glutamine, 0.5 mM L-carnitine (Sigma Aldrich), and 1% FCS), counted and then seeded in FAO assay medium [KHB (111 mM NaCl, 4.7 mM KCl, 1.25 mM CaCl_2_, 2 mM MgSO_4_, 1.2 mM NaH_2_PO_4_) supplemented with 2.5 mM glucose, 0.5 mM L-carnitine, and 5 mM HEPES, adjusted to pH 7.4 at 37 °C]. Etomoxir (40 μM) in FAO assay medium was added to control wells 15 min prior to running the assay in the Seahorse analyzer. Finally, BSA or Palmitate-BSA FAO Substrate was added to the appropriate wells immediately before running the assay. OCR was measured in real time following injections of oligomycin A, FCCP, antimycin A and rotenone, and 2-deoxyglucose.

### RT qPCR Analysis of Glycolytic Genes

Memory CD4^+^ T cells (1 × 10^6^ cells per well of 12-well plate) were left unstimulated or stimulated with anti-CD3 and−28 antibodies and treated in the presence or absence of DCA (10 mM) for 4 h. Following incubation, the cells were lysed using Qiazol lysis reagent (Qiagen) and frozen to −80 °C. RNA was isolated according to manufacturer's instructions (miRNeasy kit, Qiagen) and transcribed to cDNA using a high-capacity cDNA binding kit (Bio-Sciences). The cDNA was analyzed by RT-qPCR for the expression of Glut1, hexokinase 2 (HK2), lactate dehydrogenase (LDHa), and glucose 6 phosphate dehydrogenase (G6PD) using forward and reverse primers (Integrated DNA Technologies), relative to the housekeeper gene ribosomal protein lateral stalk subunit p0 (RPLP0). Data was normalized to the unstimulated control.

### Statistical Analysis

Statistical analyses were performed using Prism 5 software; 2 groups within a sample were determined by Student's Paired *T*-test with two-tailed *p*-values. *P*-values of < 0.05 were considered significant and denoted as follows: ^*^p < 0.05, ^**^p < 0.01, and ^***^p < 0.001.

## Results

### Th17-Lineage Cells Show Increased Expression of Glycolytic Markers Compared With Non-th17 Cells

**Figure 1:**
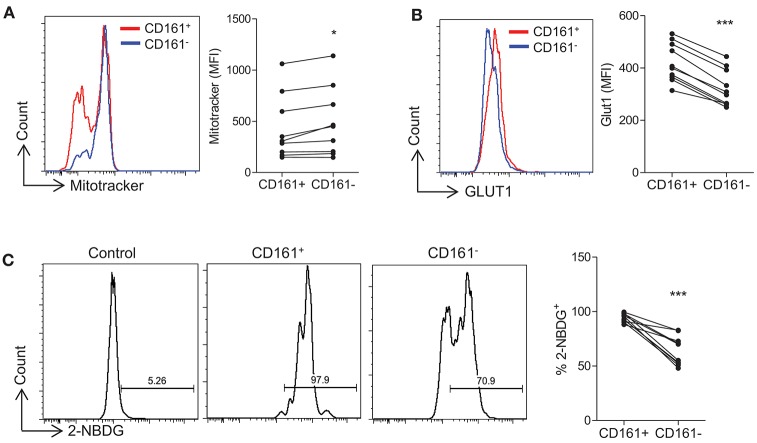
Th17-lineage cells show increased expression of glycolytic markers compared with non-Th17 cells. PBMC were isolated from healthy controls and cells were stained *ex vivo* with fluorochrome-conjugated antibodies specific for CD4, CD45RO, CD161, and MitoTracker® Green. The expression of MitoTracker® Green in CD4^+^CD45RO^+^CD161^+^ (CD161^+^) and CD4^+^CD45RO^+^CD161^−^ (CD161^−^) (*n* = 9) **(A)**. Memory CD4^+^ T cells were isolated from HC by magnetic separation and stimulated in the presence of anti-CD3 and irrAPC. Cells were stained with fluorochrome-conjugated antibodies specific for CD4, CD161, Glut1, and 2-NBDG. The expression of Glut1 in CD4^+^ CD161^+^ (CD161^+^) and CD4^+^ CD161^−^ (CD161^−^) (*n* = 10) at 24 h stimulation **(B)**. The uptake of 2-NBDG in CD161^+^ and CD161^−^ cells compared with unstimulated CD4^+^ T cells (control) (*n* = 10) at 72 h stimulation **(C)**. ^*^p < 0.05, ^***^p < 0.001.

Initially we sought to examine the presence of metabolic markers that correlate with metabolic pathways in human Th17 cells. Human PBMC were stained with MitoTracker® dye which provides an indication of mitochondrial mass, a correlate of oxidative phosphorylation. Memory CD4^+^CD161^−^ (non-Th17 lineage cells) exhibited significantly higher levels of MitoTracker® dye compared with memory CD4^+^CD161^+^ (Th17-lineage cells) (*p* < 0.05) (Figure 1A), suggesting that Th17-lineage cells may utilize less oxidative phosphorylation than non-Th17 cells. Glycolysis relies on the uptake of glucose via specific cell surface transporters such as Glut1, and the expression of Glut1 has been shown to correlate with glycolytic activity (20, 21). We therefore examined the expression of Glut1 on sorted and activated human memory CD45RO^+^CD4^+^ T cells and demonstrated significantly increased Glut1 expression on Th17 vs. non-Th17 lineage cells (*p* < 0.001) (Figure 1B). We also examined the uptake of 2-NBDG, a fluorescent glucose analog, and showed significantly increased uptake of 2-NBDG by Th17-lineage cells compared with non-Th17 lineage cells (*p* < 0.001) (Figure 1C). These data suggested that Th17-lineage cells have an increased capacity for glucose uptake, indicative of increased glycolytic activity.

### Th17-Lineage Cells Are Dependent on Glycolysis

**Figure 2:**
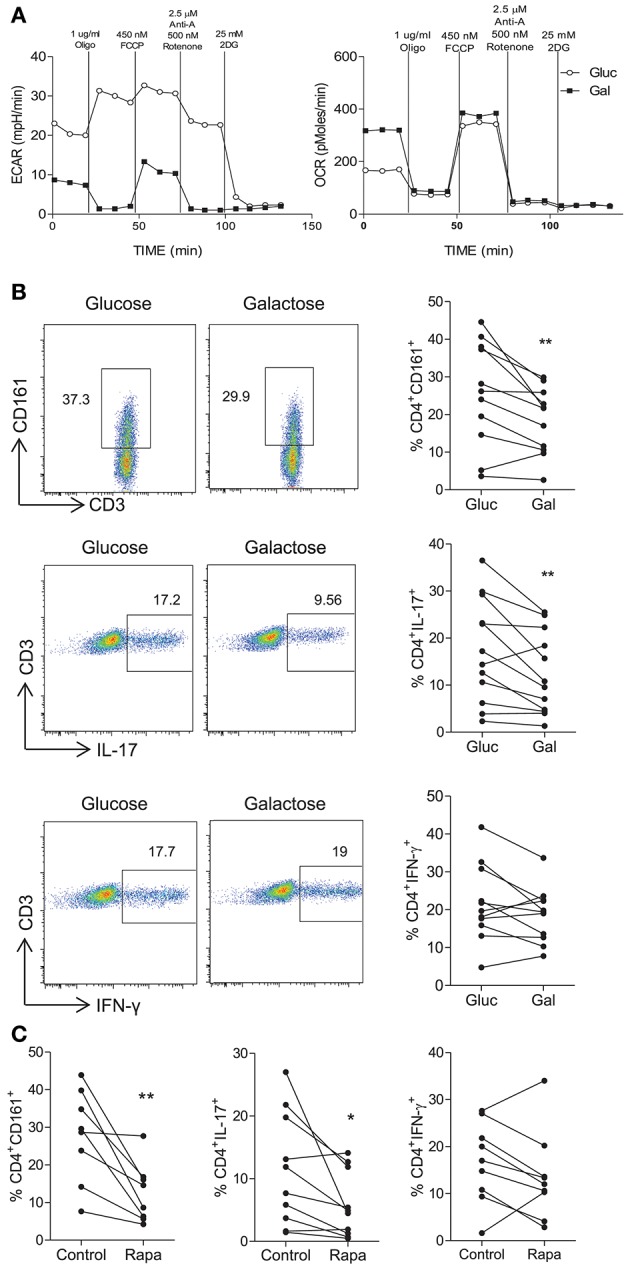
Inhibition of glycolysis constrains Th17-lineage cells. PBMC were isolated from healthy donors. CD4^+^ T cells were isolated by magnetic separation and stimulated for 18 h with PMA and ionomycin in glucose-free RPMI supplemented with glucose (10 mM) (Gluc) or galactose (10 mM) (Gal). Representative plots of ECAR and OCR over time are shown **(A)**. Memory CD45RO^+^CD4^+^ T cells were isolated by magnetic separation and cultured for 5 d in the presence of anti-CD3 and irrAPC in glucose-free medium supplemented with glucose or galactose. Cells were re-stimulated with PMA and ionomycin in the presence of brefeldin A; stained for CD161, IL-17, IFN-γ, CD3, and CD8 and analyzed by flow cytometry. Representative dot plots (gated on single, live, CD4 [CD3^+^CD8^−^] cells), accompanied by the frequencies of CD4^+^ T cells expressing CD161 and proinflammatory cytokines (*n* = 11) **(B)**. Memory CD4^+^ T cells were cultured for 5 d with anti-CD3 and irrAPC in the presence or absence of rapamycin (Rapa). Cells were re-stimulated with PMA and ionomycin in the presence of brefeldin A, stained for CD161, IL-17, IFN-γ, CD3, and CD8 and analyzed by flow cytometry. The frequencies of CD4^+^ T cells (gated on live, single CD3^+^CD8^−^ cells) expressing CD161, IL-17, or IFN-γ (*n* = 8–10) **(C)**. ^*^p < 0.05, ^**^p < 0.01.

Having demonstrated that Th17-lineage cells expressed markers consistent with a glycolytic profile, we next determined whether they were dependent on glycolysis for their function. Replacement of glucose with galactose as a fuel source is known to inhibit glycolysis (22) as confirmed in Figure 2A, where activated CD4^+^ T cells cultured in galactose containing medium exhibited reduced ECAR levels compared with those cultured in glucose containing medium, whereas OCR was unchanged except for basal OCR which was relatively increased in galactose containing medium. No differences in cell viability were observed between glucose and galactose conditions (data not shown). Having confirmed that glucose deprivation inhibits glycolysis, human CD45RO^+^CD4^+^ T cells were activated and cultured for 5 days in medium containing either glucose or galactose and their expression of CD161, IL-17, or IFN-γ was examined by flow cytometry. CD4^+^ T cells cultured in galactose exhibited significantly reduced expression of both CD161 (*p* < 0.01) and IL-17 (*p* < 0.01) by CD4^+^ T cells (Figure 2B). On the other hand, there was no significant change in the expression of IFN-γ by CD4^+^ T cells (Figure 2B). Glycolysis has been shown to be dependent on mTOR signaling (10), therefore sorted CD45RO^+^CD4^+^ T cells were stimulated for 5 d in the presence or absence of the mTOR inhibitor rapamycin. Expression of both CD161 (*p* < 0.01) and IL-17 (*p* < 0.05) by CD4^+^ T cells was significantly reduced in the presence of rapamycin (*p* < 0.05), whereas IFN-γ was unchanged (Figure 2C). As an alternative strategy to inhibit glycolysis, we also treated memory CD4^+^ T cell cultures with DCA, which directly inhibits pyruvate dehydrogenase kinase in the glycolytic pathway. As shown in Figure S1, DCA significantly reduced the frequency of Th17 cells (*p* < 0.001) (Figure S1A) in addition to their survival (*p* < 0.01) (Figure S1B) and proliferation (*p* < 0.05) (Figure S1C). In contrast, DCA had no significant effect on the frequency, viability or proliferation of Th1 cells (Figures S1A–C). The efficacy of DCA in inhibiting glycolysis was confirmed in Figure S2A, where DCA inhibited the expression of genes associated with glycolysis *HK2, LDHa, G6PD*, and *GLUT1*. Furthermore, Seahorse analysis showed that DCA reduced the ECAR and ECAR:OCR ratio in memory CD4 T cells, indicating inhibition of glycolysis (Figures S2B,C). Taken together, these data indicate that inhibition of glycolysis via glucose removal, inhibition of pyruvate dehydrogenase kinase or mTOR inhibition reduced the frequency of Th17 cells but did not appear to constrain Th1 cells, suggesting that Th17 cells are dependent on glycolysis.

### Th17 Lineage Cells Are Dependent on Fatty Acid Synthesis

**Figure 3:**
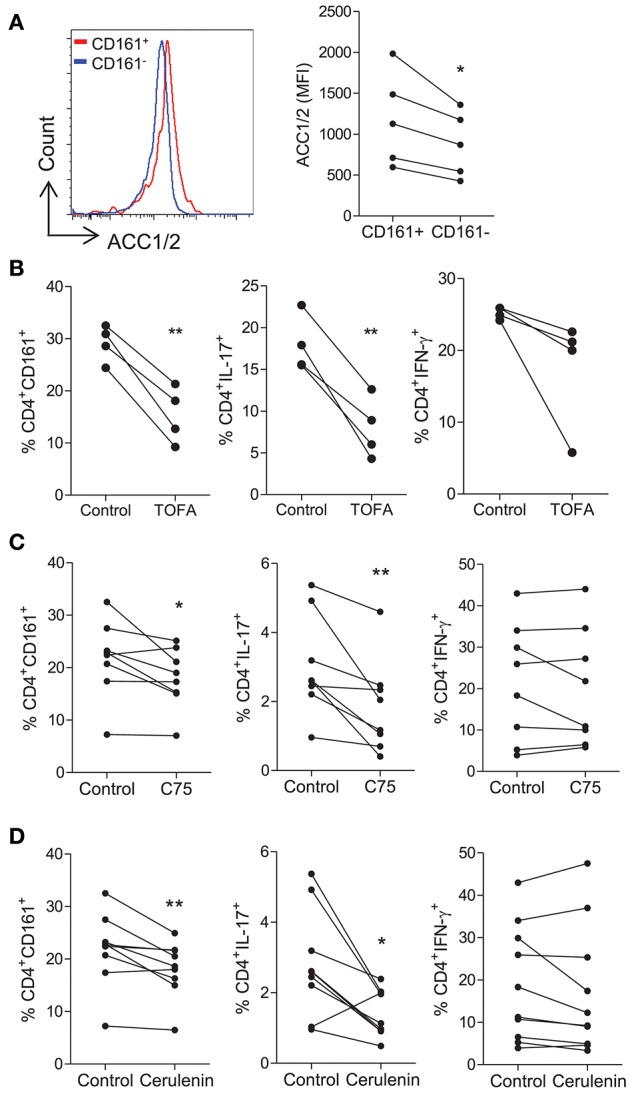
Th17-lineage cells are reduced following the inhibition of fatty acid synthesis. Memory CD45RO^+^CD4^+^ T cells were isolated from PBMC of healthy donors, stimulated with anti-CD3 and irrAPC for 5 d and stained for CD4, CD161, and ACC1/2. Representative histograms and MFI of ACC1/2 expression on CD4^+^CD161^+^ (CD161^+^) and CD4^+^CD161^−^ (CD161^−^) cells (*n* = 5) **(A)**. Memory CD45RO^+^CD4^+^ T cells were cultured for 5 d with anti-CD3 and irrAPC in the presence or absence of TOFA, C75, or cerulenin. Cells were re-stimulated with PMA and ionomycin in the presence of brefeldin A; stained for CD161, IL-17, IFN-γ, CD3, and CD8 and analyzed by flow cytometry (gated on live, single CD3^+^CD8^−^ cells). The effect of TOFA (*n* = 4) **(B)**, C75 (*n* = 8) **(C)**, or cerulenin (*n* = 9–10) **(D)** on the frequencies of CD4^+^ T cells expressing CD161, IL-17 and IFN-γ are shown. **(B)** ^*^p < 0.05, ^**^p < 0.01.

We next investigated the role of fatty acid synthesis in fuelling human CD4^+^ T cells. Initially, the expression of ACC1/2 was determined on CD161^+^ and CD161^−^ CD4^+^ T cells, since ACC1/2 enzymes are involved in the FAS pathway. Increased expression of ACC1/2 was observed on the CD4^+^CD161^+^ cells when compared with CD4^+^CD161^−^ T cells (*p* < 0.05) (Figure 3A). Next, the effect of TOFA, which is an ACC1/2 inhibitor, on CD4^+^ T cells was investigated. Memory CD45RO^+^CD4^+^ T cells were isolated and stimulated for 5 days in the presence or absence of TOFA. In cells cultured with TOFA we observed a significant decrease in the frequency of CD4^+^CD161^+^ (*p* < 0.01) and CD4^+^IL-17^+^ (*p* < 0.01) T cells (Figure 3B). In contrast however, there was no change in the frequency of CD4^+^IFN-γ^+^ T cells (Figure 3B). The effect of blocking the FAS pathway downstream of ACC1/2, by using inhibitors of FASN was investigated next. Memory CD45RO^+^CD4^+^ T cells were isolated and stimulated for 5 days in the presence or absence of the fatty acid synthase (FASN) inhibitors C75 or cerulenin. In cells cultured with C75 we observed a significant decrease in the frequency of CD4^+^CD161^+^ (*p* < 0.05) and CD4^+^IL-17^+^ (*p* < 0.01) T cells (Figure 3C). In contrast however, there was no change in the frequency of CD4^+^IFN-γ^+^ T cells (Figure 3C). As shown in Figure S3, C75 significantly inhibited the proliferation of Th17 lineage cells (*p* < 0.0001) with a lesser effect of the proliferation of Th1 cells (*p* < 0.05). Cerulenin exerted a similar effect to that of C75, showing a significant decrease in the frequency of CD4^+^CD161^+^ (*p* < 0.01) and CD4^+^IL-17^+^ (*p* < 0.05) T cells, but had no effect on the frequency of CD4^+^IFN-γ^+^ T cells (Figure 3D). Taken together, these data indicate that Th17-lineage cells utilize, and are relatively more dependent on fatty acid synthesis than Th1 cells.

### Treg Cells Exhibit Increased Oxidative Phosphorylation and Glycolysis, but Are Not Dependent on Glycolysis

**Figure 4:**
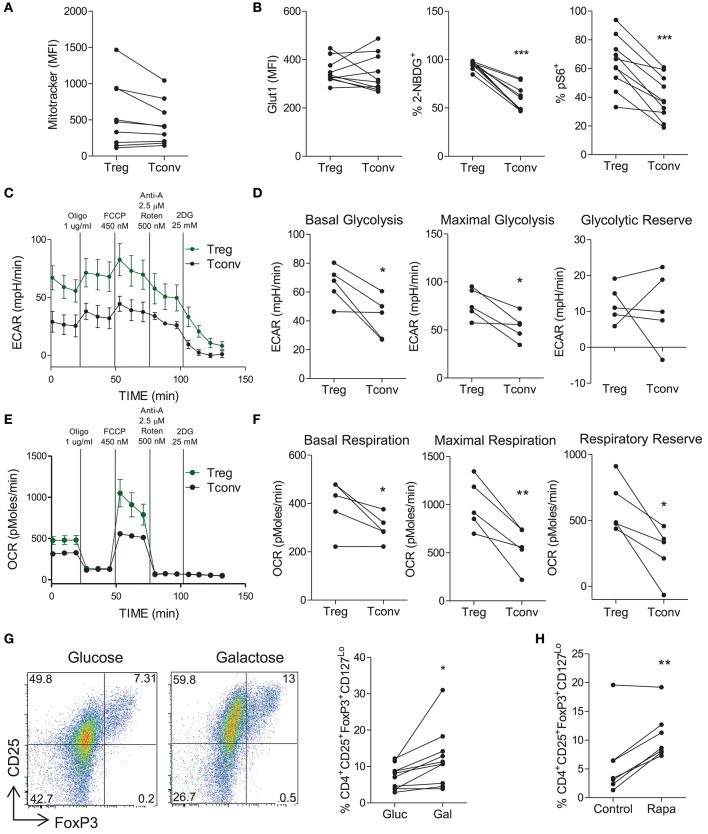
Treg cells demonstrate increased glycolysis and oxidative phosphorylation relative to conventional T cells, but do not depend on glycolysis. PBMC were isolated from healthy controls and cells were stained *ex vivo* for CD4, CD25, CD45RO, CD127 and MitoTracker® Green. MitoTracker® Green staining in CD4^+^CD45RO^+^CD25^+^CD127^Lo^ (Treg) and CD4^+^CD45RO^+^NotCD25+CD127Lo (Tconv) (*n* = 9) **(A)**. Memory CD4^+^ T cells were isolated from PBMC by magnetic separation and stimulated with anti-CD3 and irrAPC. Cells were stained for CD4, CD25, CD127, FoxP3, Glut1, pS6, and 2-NBDG uptake was measured. The expression of Glut1 in Treg and Tconv at 24 h stimulation, 2-NBDG at 72 h, and pS6 at 24 h (*n* = 10) **(B)**. Treg and Tconv cells were cell sorted from PBMC. Cells were cultured for 6 d in the presence of anti-CD3, irrAPC and IL-2. Cells were stimulated for 18 h with PMA and ionomycin prior to Seahorse extracellular flux analysis. Representative plot of ECAR over time for Treg and Tconv **(C)**. Basal glycolysis, maximal glycolytic capacity and glycolytic reserve rates for Treg and Tconv (*n* = 5) **(D)**. Representative plot of OCR over time for Treg and Tconv **(E)**. Basal respiration, maximal respiratory capacity and respiratory reserve rates for Treg and Tconv (*n* = 5) **(F)**. Memory CD4^+^ T cells were isolated by magnetic separation and cultured for 5 d in the presence of anti-CD3 and irrAPC in glucose-free medium supplemented with glucose (Gluc) or galactose (Gal). Cells were stained for CD4, CD25, CD127, and FoxP3; and analyzed by flow cytometry. Representative dot plots accompanied by the frequencies of Treg cells in glucose or galactose medium (*n* = 9) **(G)**. Memory CD4^+^ T cells were cultured for 5 d with anti-CD3 and irrAPC in the presence or absence of rapamycin (Rapa). Cells were stained for CD4, CD25, CD127, and FoxP3; and analyzed by flow cytometry. The frequencies of Treg cells following control or Rapa treatment (*n* = 6) **(H)**. ^*^p < 0.05, ^**^p < 0.01, ^***^p < 0.001.

Next the metabolic profile of Treg cells was investigated and compared to that of non-Treg (Tconv) cells. PBMC were stained for the expression of Treg cell markers and MitoTracker® Green as an indicator of mitochondrial mass. Memory Treg cells (CD4^+^CD45RO^+^CD25^+^CD127^Lo^) trended toward higher mitochondrial mass than memory Tconv cells (CD4^+^CD45RO^+^ NOT CD25^+^CD127^Lo^) (*p* = 0.07) (Figure 4A). We also examined the expression of Glut1, pS6, and uptake of 2-NBDG on Treg vs. Tconv cells. Although no difference in the expression of Glut1 between Treg and Tconv cells was observed, there was a significant increase in the uptake of 2-NBDG by Treg cells (*p* < 0.001) (Figure 4B). There was also a significant increase in the expression of pS6 in Treg cells compared with Tconv cells (*p* < 0.001), indicating increased activation of mTOR in Treg cells (Figure 4B). These data suggest that indicators of both glycolysis and oxidative phosphorylation were elevated in Treg cells when compared with Tconv cells. In order to investigate the metabolic profile of Treg cells further, we performed Seahorse flux analysis using purified Treg and Tconv cells. As shown in Figure 4C, Treg cells exhibited an overall higher ECAR profile, and significantly increased basal glycolysis (*p* < 0.05) and maximal glycolysis (*p* < 0.05) compared with Tconv cells (Figure 4D). No difference in glycolytic reserve was observed (Figure 4D). In addition, Treg cells exhibited an overall increase in OCR profile compared with Tconv cells (Figure 4E). This was reflected in significantly increased basal respiration (*p* < 0.05), maximal respiration (*p* < 0.01), and respiratory reserve (*p* < 0.05) in Treg vs. Tconv cells (Figure 4F). These data indicated that activated Treg cells utilize both glycolysis and oxidative phosphorylation to a greater extent than activated Tconv cells. Since it was possible that the expansion protocol used above prior to Seahorse flux analysis may have altered the metabolic profiles of Treg or Tconv, we also performed Seahorse analysis on freshly sorted Treg and Tconv that were stimulated for 18 h. However, as shown in Figure S4, Treg cells failed to be fully activated after 18 h stimulation with PMA/Ionomycin. Both OCR (Figure S4A) and ECAR (Figure S4B) were very low in Treg compared with Tconv. Nonetheless the ECAR:OCR ratio was higher in Treg than Tconv (Figure S4C). A relative lack of activation of Treg relative to Tconv cells was indicated by significantly lower expression of the early activation marker CD69 on Treg cells (Figure S4D). Furthermore, the fact that similar levels of activation (Figures S5A–C) and proliferation (Figure S5D) were observed after the 5 day expansion protocol validates this protocol which was used in Figure 4. Next we investigated the dependence of Treg cells on glycolysis by inhibiting glycolysis via glucose deprivation or mTOR inhibition. Culture of memory CD4^+^ T cells in the presence of galactose vs. glucose resulted in an increased frequency of Treg cells relative to Tconv cells (*p* < 0.05) (Figure 4G), as did culture with rapamycin (*p* < 0.01) (Figure 4H). We also used DCA as an alternative strategy to inhibit glycolysis, and determined the effect on the frequency of Treg cells as well as their viability and proliferation. Culture in the presence of DCA did not significantly alter the overall frequency of Treg cells although there were variable effects in different donors (Figure S1A). DCA reduced the viability (*p* < 0.05) and reduced proliferation of Treg cells in the majority of donors (ns), but not to the extent that was seen for Th17 cells (Figures S1B,C). Taken together these data indicate that activated Treg cells can utilize both glycolysis and oxidative phosphorylation. Furthermore, Treg cells are somewhat dependent on glycolysis for their proliferation but not to the same extent as Th17 cells. Hence any increased frequency of Treg cells observed when glycolysis was inhibited is the result of a relative advantage for Treg cells.

### Treg Cells Exhibit Increased Oxidation of Fatty Acids Compared With Tconv Cells and Are Not Dependent on Fatty Acid Synthesis

**Figure 5:**
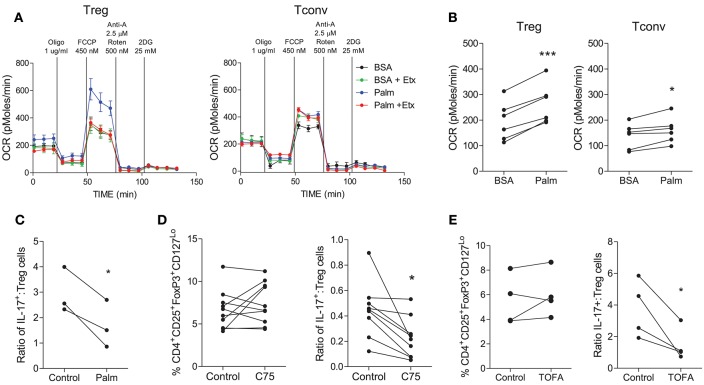
Treg cells demonstrate increased oxidation of fatty acids compared with Tconv cells and are not reliant upon fatty acid synthesis. Treg (CD4^+^CD25^+^CD127^Lo^) and Tconv (CD4^+^CD45RO^+^NotCD25^+^CD127^Lo^) cells were cell sorted from PBMC. Cells were cultured for 6 d in the presence of anti-CD3, irrAPC, and IL-2. Cells were stimulated for 18 h with PMA and ionomycin prior to Seahorse extracellular flux analysis. The Seahorse XF Palmitate-BSA FAO assay was performed to analyse the oxidation of fatty acids to fuel mitochondrial respiration. Representative plot of OCR over time for Treg and Tconv in the presence of BSA (BSA) or palmitate (Palm) and in the presence or absence of etomoxir (Etx) **(A)**. OCR for Treg and Tconv in the presence of BSA control or palmitate (*n* = 6) **(B)**. Memory CD4^+^ T cells were isolated by magnetic separation and cultured for 5 d in the presence of anti-CD3 and irrAPC in glucose-free medium supplemented with galactose in the presence or absence of palmitate (Palm). The ratio of IL-17^+^ to Treg cells is shown **(C)**. Memory CD45RO^+^CD4^+^ T cells were cultured for 5 d with anti-CD3 and irrAPC in the presence or absence of TOFA or C75 and stained directly for Treg markers CD4, CD25, CD127, and FoxP3, or re-stimulated with PMA and ionomycin and stained for IL-17, CD3, and CD8 and analyzed by flow cytometry (gated on live, single CD3^+^CD8^−^ cells). The frequencies of Treg cells, and the ratio of IL-17^+^ to Treg cells following culture with TOFA (*n* = 4) **(D)** or C75 (*n* = 9) **(E)**. ^*^p < 0.05, ^***^p < 0.001.

Next we investigated the fatty acid metabolism requirements of human Treg cells. In order to measure FAO, the Seahorse XF FAO assay was utilized where palmitate-BSA is provided as a source of FA and uptake of fatty acids is inhibited using etomoxir as an inhibitor of CPT1. In the presence of palmitate, Treg cells exhibited an increased OCR profile and this was inhibited by etomoxir (Figure 5A), indicating that Treg cells were taking up and oxidizing FA. On the other hand Tconv cells did not exhibit obviously increased OCR in the presence of palmitate (Figure 5A). Overall there was a highly significant increase in OCR from Treg cells in the presence of palmitate-BSA vs. the control BSA (*p* < 0.001), while there was a lesser increase in OCR from Tconv cells in the presence of palmitate vs. BSA control (*p* < 0.05) (Figure 5B). Having previously shown that Th17 cells relied on the synthesis of fatty acids, we next investigated the effect of promoting FAO on the Th17:Treg cell ratio. In order to exclude the confounding factor of cells utilizing glycolysis as an alternative source, we inhibited glycolysis by using galactose medium. Memory CD4^+^ T cells were stimulated and cultured in glucose free medium supplemented with galactose in order to inhibit glycolysis, and in the presence or absence of palmitate. Culture with palmitate decreased the ratio of Th17:Treg cells (*p* < 0.05), indicating that promoting FAO in the absence of glycolysis manipulates the Th17:Treg cell ratio in favor of Treg cells (Figure 5C). Finally, we investigated the role of FAS in Treg cells by culturing memory T cells in the presence or absence of the ACC1/2 inhibitor TOFA or the FASN inhibitor C75. In contrast to the previously shown dependence on FAS for Th17-lineage cells, inhibition of FASN exerted no overall effect on the frequency of Treg cells compared with control (Figure 5D). However, when the ratio of Th17:Treg cells was compared in the presence or absence of C75, there was a significant decrease in the Th17:Treg cell ratio when FASN was inhibited (*p* < 0.05) (Figure 5D). Similarly, ACC1/2 inhibition did not affect the frequency of Treg cells, but did significantly decrease the ratio of Th17:Treg cells (*p* < 0.05) (Figure 5E). Together these data indicate that inhibition of FAS or promotion of FAO modulates the Th17:Treg axis in favor of Treg cells.

## Discussion

In this study we examined the metabolic requirements of human T cell subsets and the effect of manipulating metabolic pathways on Th17 and Treg cells. As the Th17:Treg cell axis is dysregulated in a number of autoimmune or inflammatory diseases, it is therefore of key importance to identify novel strategies to modulate this axis in favor of Treg cells. We investigated the role of carbohydrate and fatty acid metabolism in the regulation of human memory T cell subsets in order to gain insight as to how effector T cells and Treg cells may be regulated at sites of inflammation. We found that Th17-lineage cells were dependent on glycolysis, as glucose-deprivation, inhibition of glycolysis, and treatment with rapamycin resulted in a reduction of these cells. Interestingly, activated Treg cells exhibited increased glycolysis, mitochondrial respiration and FAO; whereas Th17 cells demonstrated a reliance upon FAS for survival. Here we expose a fundamental difference between human Treg and Th17 cells and a possible mechanism for targeting and/or manipulating the Th17:Treg cell axis.

In this project we considered the role of metabolic pathways in regulating differentiated human memory T cells, since these are the cells that would be found at sites of autoimmune inflammation where nutrients are most likely to be limiting, requiring cells to undergo metabolic reprogramming. Murine studies have shown a clear role for metabolism in reciprocally regulating the *de novo* differentiation of Th17 and Treg cells, where differentiation of Th17 cells depended on HIF-1α and glycolysis, while Treg cell differentiation was inhibited by HIF-1α and required oxidative phosphorylation (10, 12, 23). In addition development of Th17 cells was dependent on fatty acid synthesis, whereas Treg cell differentiation required FAO (12, 18). However, recent evidence indicates that there may be important differences in the metabolic requirements for differentiation of Treg cells as opposed to activated Treg cells engaged in suppression (24). Furthermore, in the context of autoimmunity where already differentiated natural/thymically derived Treg cells control pathogenic autoreactive effector T cells, the regulation of activated Treg cell proliferation and suppressive function may be more relevant than that of Treg cell differentiation. Thus, if metabolic pathways are to be targeted to regulate T cell pathogenicity in disease then it will be important to gain a clearer understanding of how metabolic requirements impact on the effector and regulatory functions of differentiated human T cells, where the metabolic requirements are poorly understood.

We demonstrated that activated human Th17 cells required glycolysis since they were inhibited in the absence of glucose and after mTOR or pyruvate dehydrogenase kinase inhibition. Consistent with this, Th17-lineage cells exhibited increased expression of Glut1 and uptake of a glucose analog relative to non-Th17 lineage cells. On the other hand, IFN-γ producing Th1 cells were not inhibited when glycolysis was blocked. Memory CD161^−^ T helper cells (comprising mostly Th1 cells) exhibited increased mitochondrial mass relative to Th17 lineage cells, suggesting that Th17 cells are less inclined to utilize oxidative phosphorylation than other Th cells. Together these data indicated that Th17 cells utilize glycolysis and are dependent on it. Whilst there is a dearth of directly comparable studies in human T cells, murine studies have demonstrated that activation and differentiation of naïve T cells into various effector T cell subsets in general requires metabolic reprogramming and a switch to aerobic glycolysis (25). Specifically, murine Th17 cell differentiation was shown to be dependent on HIF-1α and glycolysis, with reciprocal effects on Treg cells (10). In contrast to the study by Shi et al. (10) a recent study demonstrated that various conditions that promote cellular stress, including glucose deprivation induced by 2-deoxyglucose, actually promoted Th17 cell differentiation and inhibited Th1, Th2, and Treg cell differentiation (26). It is not yet clear how this disparity can be explained. Another murine study showed that differentiation of murine Th cell subsets including Th1, Th2, and Th17 cells depended on glycolysis, whereas differentiation of Treg cells required FAO (12). The latter findings indicate that differentiation of all effector T cell subsets require glycolysis, whereas we found that committed human Th17 lineage cells depended on glycolysis, but other effector T cell subsets such as IFN-γ producing Th1 cells did not. However, in support of our findings, in a recent study the metabolic requirements of *in vitro* polarized murine Th1, Th17, and Treg cells were compared where Th17 cells exhibited the highest ECAR rate followed by Th1 cells, while Treg cells had substantially lower ECAR (27). Interestingly, PDHK1 which diverts pyruvate away from oxidative phosphorylation toward lactate, was expressed in Th17 cells but not Th1 or Treg cells (27). Thus, it would be of interest to determine whether PDHK1 expression in human Th17 cells promotes their glycolytic profile. It is possible that there may be different requirements for *in vitro* or *in vivo* differentiation, proliferation and effector functions of established T cell subsets. In support of this idea, a surprising recent finding was that differentiated murine Th17 cells generated *in vivo* were dependent on oxidative phosphorylation for their energy and cytokine production (28). Furthermore, inhibition of oxidative phosphorylation using oligomycin ameliorated Th17-driven mouse models of psoriasis and colitis (28).

We also investigated the metabolic requirements of Treg cells. Interestingly, both glycolytic and oxidative phosphorylation profiles were increased in Treg cells compared with Tconv cells, indicating flexibility in the metabolic requirements of activated Treg cells. In contrast to the findings for Th17 cells however, inhibition of glycolysis either enhanced or did not affect the frequency of Treg cells. However, this increased or unaltered Treg frequency observed after inhibition of glycolysis, appeared to result from a relative advantage conferred to Treg cells, since their proliferation was inhibited but to a lesser extent than that of Th17 cells. These data suggest that activated Treg cells utilize glycolysis as well as oxidative phosphorylation, and they are partly dependent on glycolysis. It is possible that this lesser dependence on glycolysis results from their ability to utilize oxidative phosphorylation when glycolysis is blocked. Our finding that mTOR inhibition promoted Treg cells over Tconv cells is consistent with a study which showed that rapamycin treatment favored the expansion of suppressive Treg cells, while proliferation of Tconv cells was inhibited (29). In support of our data showing that activated Treg cells utilized both glycolysis and oxidative phosphorylation, a proteomic signature of both glycolysis and FAO/oxidative phosphorylation in activated human Treg cells has been identified (30). On the other hand activated human Tconv cells exhibited a metabolic signature consistent with glycolysis (30). However, in the Procaccini et al. study, inhibition of either glycolysis or FAO inhibited the expansion of Treg cells (30), whereas we found that inhibition of glycolysis promoted the frequency of Treg cells relative to Tconv cells. This disparity may be accounted for by technical differences between the studies; the previous study used anti-leptin in addition to anti-CD3/28 stimulation in order to overcome Treg cell anergy. It is possible that the stronger stimulation may have rendered the Treg cells more dependent on glycolysis than those in our study which were stimulated with anti-CD3/28 plus IL-2. Another recent study found that human nTreg cells and tumor associated Treg cells utilized glycolysis and depended on it for their suppressive function (31). In further support of a key role for glycolysis in activated Treg cells, the relative advantage of Treg over Tconv cells in tumors was attributed to their flexibility in responding to the tumor microenvironment by utilizing glycolysis and FAS (32). In these studies (30–32), Treg cells exhibited a greater degree of dependence on glycolysis, whereas in our study Treg cells were partly dependent on glycolysis. Furthermore, the utilization of FAS by tumor Treg cells contrasts with our findings that Treg cells *in vitro* appeared to take up and oxidize FA and were not inhibited by FAS inhibitors, however it is possible that Treg cells exhibited metabolic flexibility in response to the nutrient availability within the tumor (32).

Taken together these findings challenge the dogma that Treg cells favor oxidative phosphorylation over glycolysis; and since the majority of such studies were performed in mice, it is possible that human and murine Treg cells may differ in their metabolic requirements. In addition the metabolic profiles exhibited by T cells are likely to be context specific and dependent on the activation status of the cells and availability of nutrients (33).

Human peripheral blood includes both Treg cells that originated in the thymus (tTreg) as well as those differentiated in the periphery (pTreg/iTreg). However, since it is not possible to distinguish memory tTreg from pTreg, we could not dissect the individual contributions of pTreg and tTreg to the increased glycolytic and FAO activity that we observed in Treg vs. Tconv cells. It has been shown that Treg cells induced *in vitro* by the presence of low tryptophan and high kynurenine exhibited increased glycolysis and oxidative phosphorylation relative to T effector cells and tTreg cells (34). In support of this, another study showed that the induction and function of *in vitro* differentiated pTreg was dependent on glycolysis, and that the glycolytic enzyme enolase regulated *FOXP3* splicing (35). Thus, it is possible that the increased Treg cell glycolysis and oxidative phosphorylation that we observed may be accounted for by the pTreg component of the Treg population and hence will require further investigation. Our data, showing partial dependence of Treg cell proliferation on glycolysis, taken together with other studies, call into question the wisdom of strategies to target glycolysis in Th17 driven inflammatory disease. Although inhibition of glycolysis conferred a relative advantage to Tregs in terms of frequency, an overall reduction in Treg cell proliferation and number could be disadvantageous. It has also not yet been determined how glycolytic inhibition would impact on Treg suppressive function which is an important consideration for future studies.

Lipid metabolism is another important pathway by which immune cells can potentially be modulated. We found that human Th17 cells were dependent on FAS for their lipid requirements since they were inhibited in the presence of an ACC1/2 inhibitor and two different FASN inhibitors. Consistent with this finding, Th17 cells expressed higher levels of the ACC1/2 enzymes involved in the FAS pathway. In contrast however, Treg cells were not inhibited by ACC1/2 or FASN inhibitors. Furthermore, ACC1/2 or FASN inhibition significantly decreased the Th17:Treg ratio, suggesting that FAS could be targeted to modulate this axis in favor of Treg cells. Rather than synthesizing fatty acids, Treg cells oxidized exogenous fatty acids as shown by Seahorse analysis of oxygen consumption, where Treg cells increased their consumption to a greater extent than Tconv cells in the presence of added palmitate. In addition, culturing memory Th cells in the presence of palmitate while blocking glycolysis modulated the Th17:Treg axis in favor of Treg cells. Of interest, a recent study using CPT1a deficient mice, has shown that murine Treg cells *in vivo* did not depend on FAO, since Treg homeostasis and suppressive function were not affected in these mice (36). It is not yet clear whether these findings extrapolate to human Treg cells, since conclusions drawn from previous studies using etomoxir (>100 μM) to inhibit CPT1a are likely to be compromised as a result of off-target effects of etomoxir (36). Our study indicates that human Treg cells oxidize fatty acids in a CPT1a dependent manner when exogenous palmitate is provided, but does not provide evidence of Treg dependence on FAO. It is possible that such a dependence on FAO may only be evident in the absence of glycolysis as an alternative energy source.

Thus, our data suggest that human Th17 lineage cells are dependent on FAS, whereas Treg cells are dependent on the uptake and oxidation of fatty acids which feed into oxidative phosphorylation. These findings are broadly consistent with other studies performed using mice, although these mostly examined the role of fatty acid metabolism in the differentiation of Th cells whereas we show effects on CD4^+^ T cells that have already differentiated into subsets. Inhibition of ACC1, which is required for FAS, inhibited the *in vitro* differentiation of Th17 cells and promoted the differentiation of Treg cells from naïve T cells, in both mice and humans (18). In addition, T cell-specific deletion of ACC1 inhibited Th17-mediated EAE, and since Th17 cells are likely to exert their effects during the induction phase of EAE, these effects were likely mediated by reduced differentiation of Th17 cells as a result of FAS inhibition (18). In addition, blockade of FASN, which is downstream of ACC1, inhibited the development of Th17 cells, and Th17-specific inhibition of FASN ameliorated EAE (37). In another murine study, enhancing FAO via AMPK activation using the AMP analog 5-aminoimidazole-4-carboxamide ribonucleotide (AICAR), promoted the polarization of Treg cells and inhibited Th17 cells both *in vitro* and *in vivo* (38). Interestingly, it was suggested that obesity induces the expression of ACC1 which drives Th17 cell differentiation via RORγt, as shown in mice fed a high fat diet (19). In addition, there was a correlation between expression of ACC1 and the frequency of Th17 cells in obese humans, suggesting that ACC1 also drives Th17 differentiation *in vivo* in humans (19). FAS was also implicated in T cell pathogenicity in RA, where blockade of FAS inhibited the tissue invasiveness of RA T cells *in vivo* (39). Together with our findings, these studies provide further support for the strategy of targeting Th17 cells via the FAS pathway on which they depend, although further investigation will be required to determine whether these findings can be extrapolated to disease settings *in vivo*.

In summary, our data indicates that inhibition of either glycolysis or FAS can tilt the Th17:Treg axis in favor of Treg cells, suggesting that these pathways could be targeted to combat multiple inflammatory diseases. However, as discussed above, the concept of a clear dichotomy between the requirements for glycolysis vs. oxidative phosphorylation by Treg and Th17 cells may be too simplistic and therefore targeting of the glycolytic pathway in inflammatory disease may not be viable. Fatty acid metabolism however, represents a promising target for the modulation of the human Th17:Treg cell axis, since inhibition of FAS or induction of FAO tips the axis in favor of Treg cells.

## Author Contributions

DC and JF contributed to the conception and design of the study. DC, AP, and BM designed and performed experiments. DC and JF wrote the first draft of the manuscript. All authors contributed to manuscript revision, read and approved the submitted version.

### Conflict of Interest Statement

This study was part funded by AbbVie Inc. JF has received honoraria from Novartis. The remaining authors declare that the research was conducted in the absence of any commercial or financial relationships that could be construed as a potential conflict of interest.

## Abbreviations

2-NBDG, 2-[*N*-(7-Nitrobenz-2-oxa-1: 3-diazol-4-yl)amino]-2-deoxy-D-glucose
ACC: Acetyl-CoA carboxylase
AICAR: 5-Aminoimidazole-4-carboxamide ribonucleotide
AMPK: AMP-activated protein kinase
CPT1: carnitine palmitoyltransferase I
EAE: experimental autoimmune encephalomyelitis
ECAR: extracellular acidification rate
FA: fatty acid
FAO: fatty acid oxidation
FAS: fatty acid synthesis
FASN: fatty acid synthase
HC: healthy control
HIF-1α: hypoxia-inducible factor 1α
Glut1: glucose transporter 1
MFI: median fluorescent intensity
mTOR: mammalian target or rapamycin
PDHK1: pyruvate dehydrogenase kinase 1
pS6: phosphorylated ribosomal protein S6
OCR: oxygen consumption rate.
