# Supplementary material for: Differential Regulation of Human Treg and Th17 Cells by Fatty Acid Synthesis and Glycolysis
Source: Front Immunol. 2019 Feb 4;10:115. doi: 10.3389/fimmu.2019.00115 (PMC6369198; doi:10.3389/fimmu.2019.00115)
Supplement: Supplementary file 1 [file Image_1.pdf]

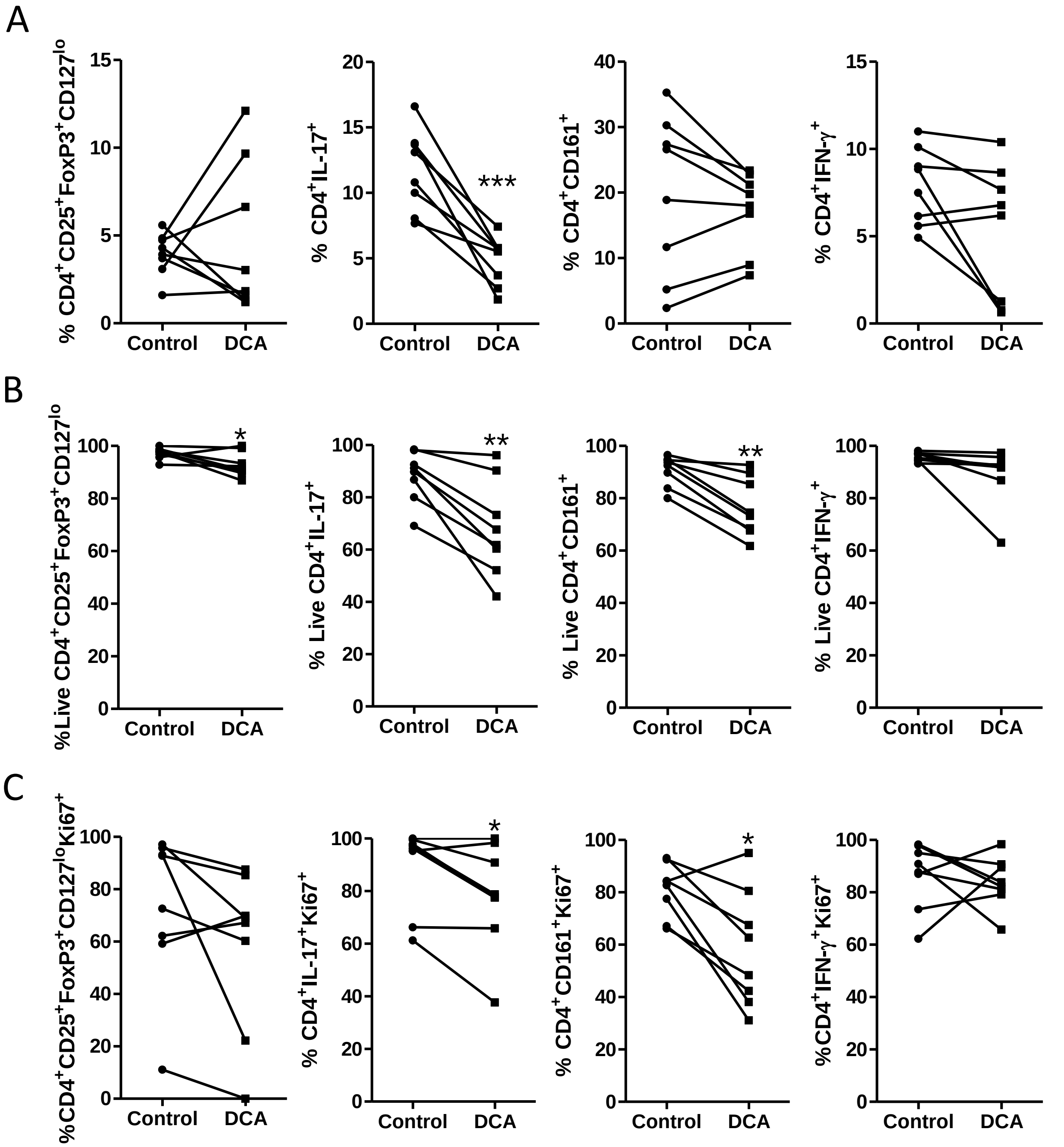

Supplemental Figure S1. The effect of inhibiting glycolysis with DCA on Treg, Th17 and Th1 cells.

Memory CD4+CD45RO+ cells were MACS sorted, stimulated with anti-CD3 plus irradiated APC for 5 days in the presence or absence of DCA. Cells were stained for Treg markers (CD4, CD127lo, CD25, FoxP3) or re-stimulated for 5 hr with PMA/Ionomycin and brefeldin A, and stained for expression of CD3, CD8, CD161, IFN-γ and IL-17 and analysed by flow cytometry (n=8). The frequency (A), viability (B) and frequency of proliferating (Ki67+) (C) Treg, Th17 (CD4+IL-17+), Th17 lineage (CD4+CD161+), or Th1 (CD4+IFN-γ+) cells are shown in the presence or absence of DCA.

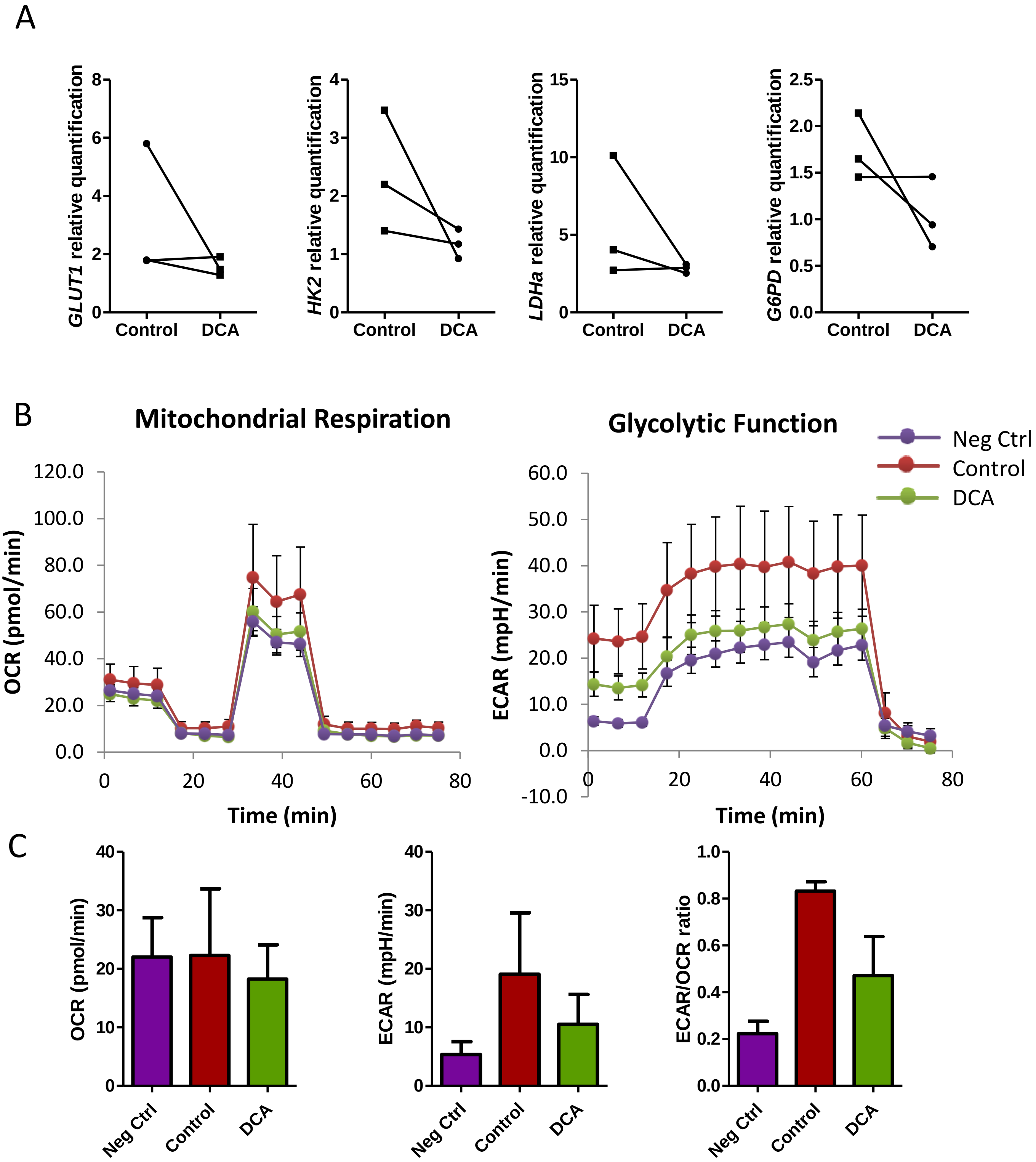

Supplemental Figure S2. The effect of DCA on the expression of glycolytic genes and metabolic profiles in memory CD4$^+$ T cells.

Memory CD4+CD45RO+ cells were MACS sorted, stimulated with anti-CD3/anti-CD28 in the presence or absence of 10 mM DCA for 4 hr (n=3). RNA was isolated, reverse transcribed into cDNA and then analysed by real time RT-PCR for the expression of Glut1, hexokinase 2 (HK2), lactate dehydrogenase (LDHa) and glucose 6 phosphate dehydrogenase (G6PD) relative to the house-keeping gene RPLP0 and normalised to the unstimulated control (A). CD4+CD45RO+ T cells were sorted and pre-treated with 10 mM DCA (DCA), left unstimulated (Neg Ctrl) or stimulated for 18 hr with PMA/Ionomycin (Control) and then analysed by Seahorse flux (n=3). Seahorse profiles of OCR and ECAR are shown in (B) and the basal OCR, ECAR and ECAR:OCR ratio are shown in (C).

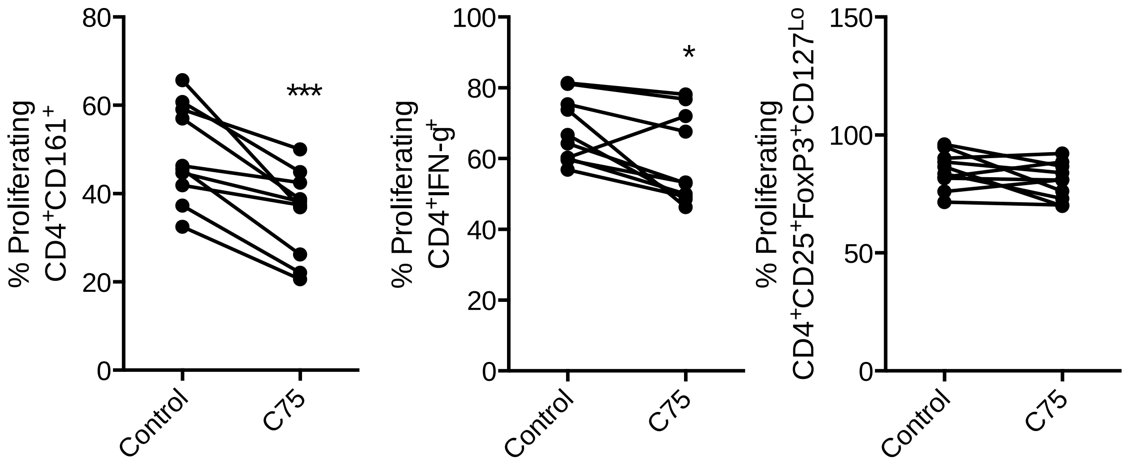

Figure S3. The effect of inhibiting FASN on Treg, Th17 and Th1 cell proliferation.
Memory CD4+CD45RO+ cells were MACS sorted and stained with CTV, stimulated with anti-CD3 plus irradiated APC for 6 days in the presence or absence of C75. Cells were stained for Treg markers (CD4, CD127lo, CD25, FoxP3) or re-stimulated for 5 hr with PMA/Ionomycin plus brefeldin A, and stained for expression of CD3, CD8, CD161 and IFN-γ and analysed by flow cytometry (n=9). The frequency or frequency of proliferated (CTVlo) Th17 lineage (CD4+CD161+), Th1 (CD4+IFN-γ+) and Treg cells are shown in the presence or absence of C75.

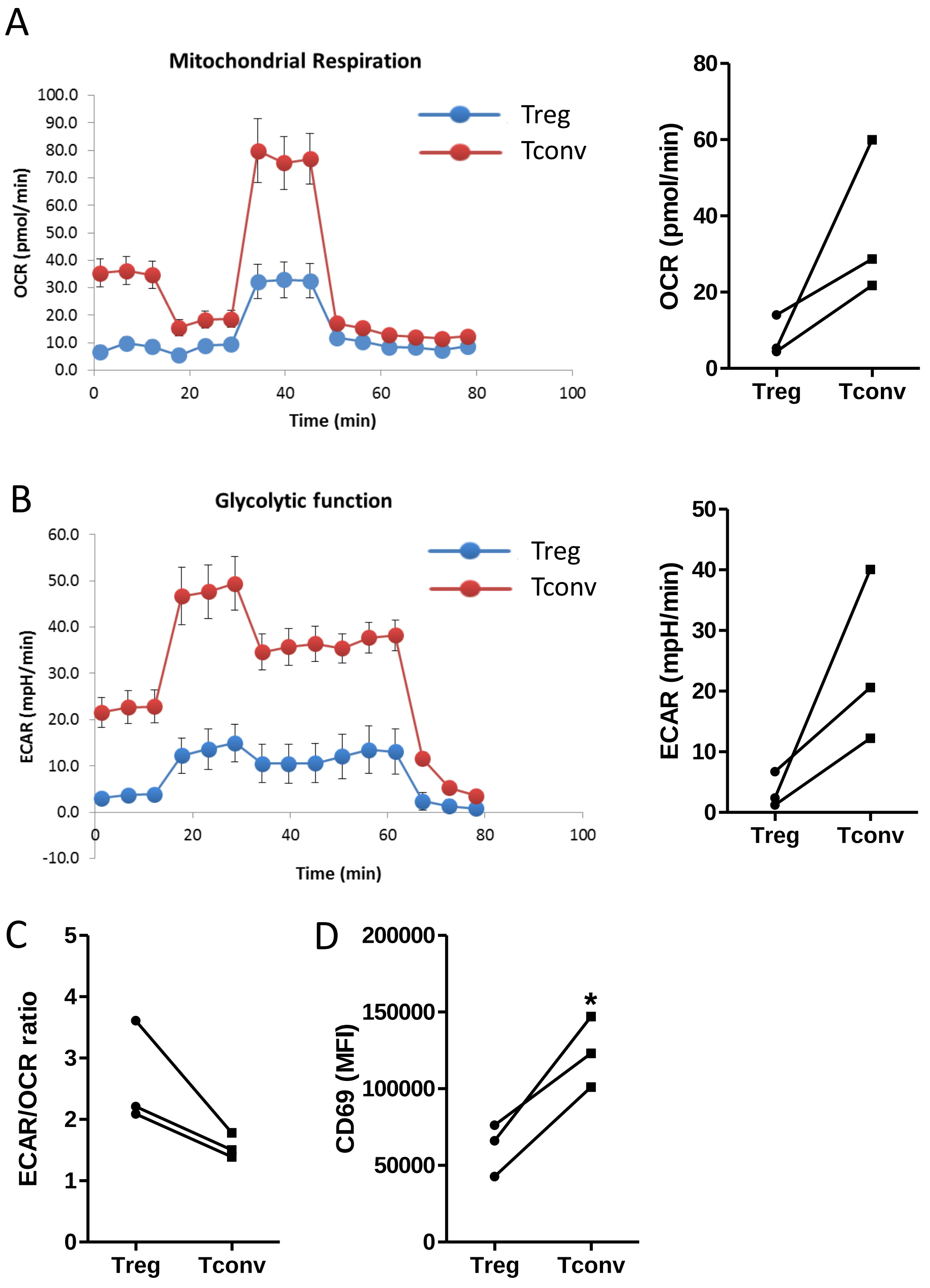

Figure S4. Seahorse analysis of Treg and Tconv cells after 18 hr stimulation. Treg (CD4+CD45RO+CD25+CD127lo) and Tconv (CD4+CD45RO+CD25-CD127+) were FACS sorted, stimulated for 18 hr with PMA/Ionomycin and then analysed by Seahorse flux (n=3). OCR (A) and ECAR (B) Seahorse profiles and basal rates are shown. The ratio of ECAR/OCR is shown in (C). Sorted Treg and Tconv were stimulated for 18 hr with PMA/Ionomycin, stained for expression of CD69 and then analysed by flow cytometry (n=3) (D). * $p<0.05$, paired t-test.

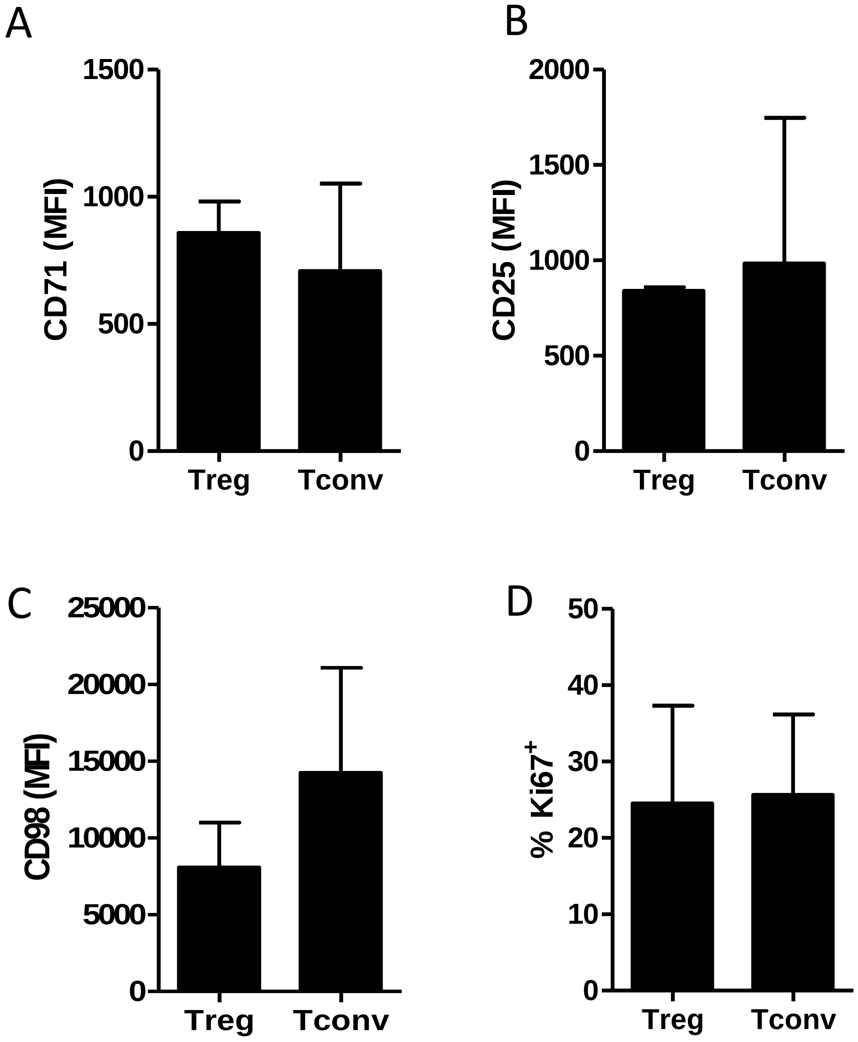

Figure S5. Activation and proliferation of Treg vs Tconv after 6 days.
Treg (CD4+CD45RO+CD25+CD127lo) and Tconv (CD4+CD45RO+CD25-CD127+) were FACS sorted, stimulated using anti-CD3/CD28 beads plus IL-2 (Treg) or anti-CD3/CD28 plus irradiated APC (Tconv). After 6 days cells were stained for expression of activation markers CD71 (A), CD25 (B), CD98 (C) or intracellular Ki67 (D) and analysed by flow cytometry (n=2).
